# Physiological Consequences of Nonsense-Mediated Decay and Its Role in Adaptive Responses

**DOI:** 10.3390/biomedicines12051110

**Published:** 2024-05-16

**Authors:** Zhengxin Ma, Ratna Sharma, Aric N. Rogers

**Affiliations:** 1 MDI Biological Laboratory, Bar Harbor, ME 04609, USA; 2 Department of Biomedical Engineering, Columbia University, New York, NY 10027, USA; rs4241@columbia.edu

**Keywords:** alternative splicing, nonsense-mediated decay, adaptive response, physiological responses

## Abstract

The evolutionarily conserved nonsense-mediated mRNA decay (NMD) pathway is a quality control mechanism that degrades aberrant mRNA containing one or more premature termination codons (PTCs). Recent discoveries indicate that NMD also differentially regulates mRNA from wild-type protein-coding genes despite lacking PTCs. Together with studies showing that NMD is involved in development and adaptive responses that influence health and longevity, these findings point to an expanded role of NMD that adds a new layer of complexity in the post-transcriptional regulation of gene expression. However, the extent of its control, whether different types of NMD play different roles, and the resulting physiological outcomes remain unclear and need further elucidation. Here, we review different branches of NMD and what is known of the physiological outcomes associated with this type of regulation. We identify significant gaps in the understanding of this process and the utility of genetic tools in accelerating progress in this area.

## 1. Introduction

Eukaryotic cells have multiple mRNA surveillance mechanisms to ensure proper protein production. One of these mRNA surveillance pathways is nonsense-mediated decay (NMD), which recognizes and eliminates mRNA containing one or more premature termination codons (PTCs). PTC-containing mRNA can lead to the production of non-functional or deleterious proteins. NMD plays a fundamental role in maintaining cellular homeostasis by regulating gene expression, controlling mRNA abundance, and modulating protein diversity. Although first recognized for its ability to surveil mRNA as a quality control mechanism to maintain the integrity and accuracy of gene expression, more recently, its broader role in regulating gene expression was recognized [1]. Its intricate regulatory pathways and mechanisms have profound implications for a wide range of biological processes, including development, disease, immune response, and stress adaptation. Understanding the complexities of NMD not only advances our knowledge of basic cellular biology, but also holds promise for developing targeted therapeutic interventions for NMD-related disorders and optimizing gene expression in various biological contexts.

In this review, we discuss the recent advances in understanding the mechanisms governing NMD and what is known about the role of particular NMD factors. Although NMD has been investigated both in vitro and in vivo, limited information exists linking NMD with physiological responses, which are made more important given our nascent understanding of its role in mediating adaptive responses and the consequences associated with altered NMD function. In addition, we discuss what models are available to further explore NMD, especially its relationship with physiological adaptation.

## 2. NMD Pathways

**Figure 1:**
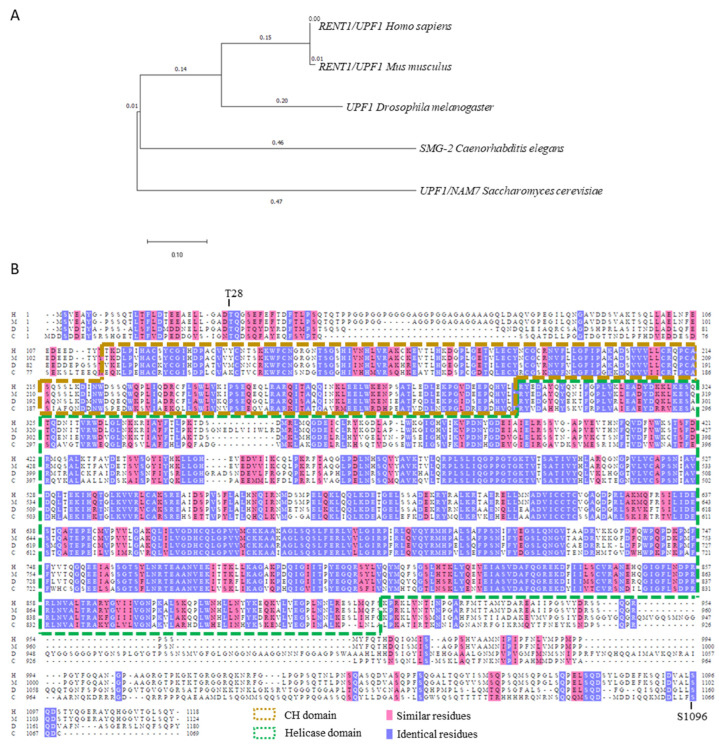
UPF1 is conserved across eukaryotic species. (**A**) Phylogenetic tree of the UPF1/SMG-2/NAM7 homologs. Sequences from *Homo sapiens*, *Mus musculus*, *Drosophila melanogaster*, *Caenorhabditis elegans*, and *Saccharomyces cerevisiae* are shown in the tree. (**B**) Alignment of UPF1 in human (H), mouse (M), fruit fly (D), and *C. elegans* (C). The brown box corresponds to the cysteine-/histidine-rich domain (CH domain), which binds to UPF2 and eRF3; the green box corresponds to the helicase domain. T28 is the phosphorylation site important for SMG6 binding to UPF1. S1096 is the phosphorylation site important for SMG5:SMG7 complex.

NMD is conserved across all eukaryotes and requires translation to identify its mRNA targets. The central factor, UPF1, has RNA-dependent ATPase and 3′-to-5′ helicase activity and is required in all eukaryotic NMD [2,3]. UPF1 is also known as NAM7 in fungi, SMG-2 in *Caenorhabditis elegans*, and RENT1 in mammals (Figure 1). The mechanisms of NMD are mostly studied using mammalian cell lines and in vitro assays, but also in vivo using *Saccharomyces cerevisiae*, *C. elegans*, and *Drosophila melanogaster* systems. Over the years, several branches of NMD were discovered based on how different mRNA substrates are targeted and the factors involved. The classical branch relies on the placement of the exon junction complex (EJC), termed EJC-dependent NMD. There is also an EJC-independent branch and one driven by alternative splicing (AS), referred to as regulated unproductive splicing and translation (RUST), or AS-NMD. Based on factors that are recruited to the pathway, other branches including UPF2-independent, UPF3-independent, and SMG1-independent NMD, have been identified [4,5]. Here, we focus on the first three branches, for which more information is available.

### 2.1. EJC-Dependent NMD

**Figure 2:**
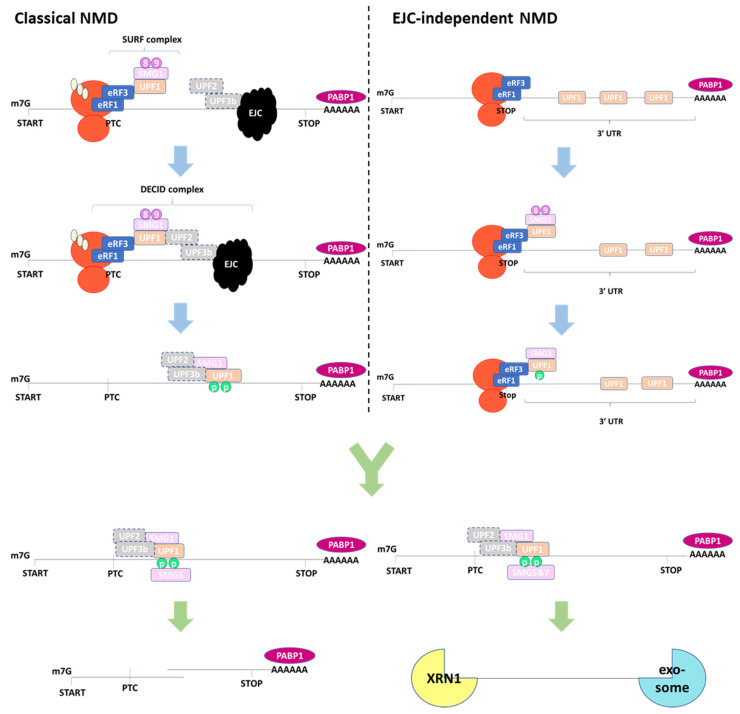
Exon junction complex (EJC)-dependent and -independent nonsense-mediated mRNA decay (NMD) mechanisms. In the classical model, UPF1, SMG1 complex, eRF1, and eRF3 form the SURF complex. Subsequently, the SURF complex binds to UPF2, UPF3b, and the EJC proteins to form the decay-inducing (DECID) complex. UPF1 is phosphorylated and activated by SMG1. In the EJC-independent model, NMD activation is triggered by the presence of a long 3′UTR. In both cases, phosphorylated UPF1 recruits either SMG6 or the SMG5:SMG7 complex to initiate endonucleolytic or exonucleolytic mRNA decay, respectively. In the figure, the illustration of SMG6 and SMG5:SMG7 recruitment and transcript degradations follow classical NMD. In EJC-independent NMD, UPF2 and UPF3b should not exist. The dashed boxes indicate that there are UPF2- and UPF3b-independent NMD pathways.

EJC-dependent NMD is the most well characterized branch (Figure 2) [1,6]. In mammalian cells, SMG1 kinase together with SMG8 and SMG9 form the SMG1 Complex (SMG1C) [7]. UPF1 and SMG1C interact with translation elongation release factors eRF1 and eRF3 to form the surveillance complex (SURF) near the PTC. SMG1C kinase activity is suppressed until the ribosome, in association with the SURF complex, reaches the EJC downstream of the PTC [7]. The SURF complex, UPF2, UPF3b, and the EJC form the decay-inducing complex (DECID), which triggers UPF1 phosphorylation by SMG1 and dissociation of release factors and the ribosome. Subsequently, UPF1 helicase activity is activated through association with UPF2. The NMD complex then moves through the EJC toward the 3′ end, at which point UPF1 binds to either the SMG5:SMG7 complex or SMG6 [1]. SMG5 and SMG7 can form a heterodimer and rapidly initiate Dcp2-dependent decapping and XRN1-dependent 5′-to-3′ mRNA degradation. SMG6 is an endonuclease that cleaves mRNA in the vicinity of the PTC [8]. The SMG5-SMG7 heterodimer and SMG6 endonuclease mediate UPF1 dephosphorylation, likely via the recruitment of phosphatase 2A (PP2A) [9,10,11]. Dephosphorylation is required before UPF1 can repeat NMD of the next target mRNA.

One question that remains unclear is why NMD harbors two mRNA decay mechanisms (via either SMG6 or SMG5:SMG7 complex) and how is it decided which RNA degrading factors to recruit. It is suggested that the phosphorylation of UPF1 at different residues results in different recruitment. The phosphorylated residue T28 prefers binding to SMG6, whereas phosphorylated site S1096 recruits SMG5:SMG7 [12] (Figure 1B). However, UPF1 has more than two phosphorylation sites and whether the phosphorylation of the other sites is involved in SMG factor recruitment remains unknown. Furthermore, it is not addressed whether SMG6- and SMG7-mediated RNA degradation target the same or distinct populations of mRNA. SMG5, SMG6, and SMG7 share similar structures. They all contain a TPR domain that consists of the 14-3-3 and helical hairpins domain [13]. Colombo et al. demonstrated a high level of redundancy in the targets of human SMG6- and SMG7-mediated decay [14]. In their study, the authors conducted RNA sequencing on HeLa cells that performed shRNA-mediated knockdown of SMG6, SMG7, or both. When SMG7 was knocked down, SMG6 mRNA was upregulated by 77% and compensated for the activity of SMG7. However, when SMG6 was absent, SMG7 was less upregulated. Nevertheless, these two factors acted on highly redundant targets and these targets include a significant portion of snoRNA and miRNA, which is consistent with previous research [15]. However, evidence suggests that, regardless of the high target redundancy, SMG6 and SMG7 have unique functions that cannot be compensated by each other. In *C. elegans*, knockdown or knockout of SMG-6 or SMG-7 independently decreased lifespan under dietary restriction (DR) [16]. In *Arabidopsis thaliana*, SMG7 is necessary to exit from meiosis and its disruption leads to embryonic lethality [17]. These studies revealed that the roles of SMG6 and SMG7 are not entirely interchangeable, and the specific functions distinguishing them remain unclear. The elusive nature of the essential functions of SMG6 and SMG7 may stem from their association with low-expressed targeted RNA, specific gene isoforms, or cell-specific NMD efficiency. Bulk and single-cell RNA sequencing with very high coverage are needed to answer this question.

It is noteworthy that some NMD factors function not only in NMD, but also in other mRNA surveillance regulatory pathways. They can even have roles outside of mRNA regulation. The NMD key factor, UPF1, is involved in replication-dependent histone mRNA decay, glucocorticoid receptor-mediated decay, regnase-1-mediated decay, and Staufen-mediated mRNA decay [18]. In addition, UPF1 has nuclear functions in telomere stability [19], DNA replication, and S phase progression [20]. These additional roles of UPF1 are independent from NMD. Thus, it is possible that the loss of this factor leads to lethality due to non-NMD functions. Similarly, some other factors of NMD also have non-NMD functions. The human SMG1 phosphorylates not only UPF1, but also p53 protein, a tumor suppressor, under genotoxic stress, indicating that SMG1 is involved in NMD-independent responses to DNA and RNA damage [21]. Via immunoprecipitation assays, human SMG5 and SMG6 were found to interact with telomerase activity [22,23], suggesting their roles in the regulation of telomere stability.

### 2.2. EJC-Independent NMD

The process by which NMD targets mRNA without an EJC is less well understood (Figure 2). This branch of NMD targets aberrant transcripts with no introns downstream of the termination codon, which cannot form an EJC. In *S. cerevisiae*, only a small fraction of transcripts are spliced [24], but about 50% of transcripts are NMD targets [15], indicating the main NMD branch in *S. cerevisiae* is EJC-independent. Sometimes referred to as long 3′UTR-mediated NMD, this branch is also observed in nematodes and mammals [1]. In the EJC-independent model, experiments indicate that mRNA with a long 3′UTR places polyadenylate-binding protein 1 (PABPC1) too far from the termination codon to efficiently recruit ribosome release factors eRF1 and eRF3, which triggers NMD activation [25]. In addition, the binding of UPF1 within long 3′UTRs increases the chance that it will be phosphorylated [26,27]. Such events lead to aberrant translation termination and NMD activation.

### 2.3. AS-NMD

AS-NMD is highly conserved in invertebrates, plants, and mammals [28,29,30,31]. The interplay between AS and NMD provides a sophisticated layer of gene regulation, influencing transcriptome diversity, protein isoform expression, and cellular functions. The process of AS allows pre-mRNA to be spliced in different ways to generate diverse mRNA products and thereby enrich protein diversity. As a surveillance mechanism, NMD targets mRNA with one or more PTCs due to mutation or unproductive splicing. In Morrison et al. [32] and subsequent studies, evidence shows that AS and NMD are coupled to regulate gene expression [31,33]. AS activates NMD by generating mRNA products with PTCs via frameshifting exons, intron retention, or exon skipping [34]. These PTC-bearing mRNAs trigger NMD to degrade them, preventing the translation of truncated or aberrant proteins. Interestingly, many of the RNA splicing factors are observed to autoregulate themselves via negative feedback loops [35]. For instance, the regulation of splicing factors *SFRS2, SFRS3,* and *SFRS7,* as well as numerous ribosomal proteins, through AS-NMD is facilitated by their corresponding proteins [36,37,38,39,40]. Jangi et al. revealed that the autoregulation of splicing factors is mediated via AS-NMD, involving cross-regulation with Rbfox2 (RNA binding FOX-1 homolog 2) [41]. These negative-feedback loops restrict expression and prevent excessive accumulation. The varying intensity of the feedback reflects the dynamic response of the system to the changing environment.

## 3. Tissue-Specific NMD Efficiency Variation

NMD efficiency is affected by many factors: targeted transcripts, PTC position in the transcripts, cell and tissue types, and cellular environment. The variation in NMD efficiency in different tissues is closely related to genetic diseases. In two human patients, PTC mutation resulted in the complete degradation of the mRNA by NMD in cartilage, but not in lymphoblasts or bone cells [42]. In human cell lines, it was found that NMD was highly efficient in HeLa cells but not in MCF7 cells (a breast cancer cell line) [43]. Similarly, via single-cell analysis, Sato and Singer provided a detailed characterization of NMD efficiency in individual cells [44]. While 72.1% of tested cells exhibited robust NMD, 13.5% of cells displayed transcripts that evaded NMD nearly entirely. Similarly, NMD efficiency across different tissues was investigated in mice [45]. In heterozygous *MenI* mice, a PTC was introduced to exon 4 of *MenI.* In this mouse model, tissues including testis, ovary, brain, and heart showed that the PTC-containing transcript was degraded more significantly (18% mutant compared to wild type *MenI* transcripts). Another group of tissues consisting of lung, intestine, and thymus had less NMD activity (35% *MenI* transcripts compared to wild type). In *C. elegans*, although NMD efficiency in different tissues was not tested directly, the expression of key NMD factors UPF1 and SMG1 are the highest in nervous, reproductive, and muscular systems, whereas the expression in epithelial tissue is very low (https://worm.princeton.edu, accessed on 10 February 2024). The complexity of NMD efficiency variation across different tissues highlights the intricate regulatory mechanisms of RNA surveillance. This variability has direct implications for understanding the pathogenesis of genetic diseases and developing targeted therapeutic interventions. Further research into the tissue-specific regulation of NMD is essential for advancing our knowledge of cellular biology and improving strategies for managing genetic disorders. For this purpose, *C. elegans* can serve as a powerful model organism, given the ready availability of tissue-specific RNA interference strains [46] and the ease of monitoring their physiological responses. In the next section, we look at the physiological consequences of normal and defective NMD in development, stress adaptation, and aging.

## 4. Physiological Consequences of NMD

Exploring the physiological consequences of NMD unveils a profound layer of cellular regulation with far-reaching implications. NMD is a crucial quality control mechanism that safeguards cells against the production of aberrant proteins and regulates normal gene expressions. Understanding the phenotypes influenced by NMD provides valuable insights into the intricate mechanisms governing biological systems. This section delves into the diverse physiological consequences of NMD, shedding light on its multifaceted roles in maintaining cellular homeostasis, shaping organism growth and health, and responding to stress (Table 1).

### 4.1. Growth and Development

NMD has important roles in the growth and development of organisms across species. In *S. cerevisiae*, NMD mutants have defective respiratory competence partially caused by the overexpression of ADR1, leading to slower growth on nonfermentable carbon sources [47]. The mutations of NMD factors in *C. elegans* cause minor developmental defects, with animals having reduced offspring numbers and abnormal morphology of genitalia [53]. Though not fatal in yeast or *C. elegans*, knockout of UPF1 is lethal at the embryonic stage in mice [61] and during larval development in *Drosophila melanogaster* [59]. Similar to UPF1, factors including UPF2, SMG1, and SMG6 are essential for mammalian embryonic viability [62,63,64]. Bao et al. reported that the depletion of *Upf2* in murine embryonic Sertoli cells caused testicular atrophy and male sterility [65]. Likewise, Wittkopp et al. showed that the depletion of UPF1, UPF2, SMG5, or SMG6 severely reduce the viability of zebrafish embryos [60]. Moreover, the composition of NMD machinery is particularly important for tissue-specific differentiation. In human early cell development, the downregulation of NMD is needed to allow cell differentiation [69]. Taken together, the observations in various model organisms, from yeast to mice, highlight the evolutionary conservation and fundamental importance of NMD in ensuring normal growth, development, and viability. 

### 4.2. Disease

A range of human diseases are associated with NMD and most of them are associated with developmental defects. It is estimated that PTCs are responsible for about 33% of inherited and acquired diseases [80]. Studies showed that Upf3b-dependent NMD regulates the development of neural cells and that loss-of-function mutation leads to intellectual disability, autism, childhood onset schizophrenia, and attention deficit hyperactivity disorder [70,71]. Similarly, heterozygous deletions of UPF2, which directly interacts with UPF3B, cause intellectual disability [72]. Homozygous loss-of-function mutations of SMG9 result in multiple congenital anomaly syndromes in humans, as it is a key component associated with UPF1 phosphorylation and is essential for normal development [67]. Similarly, Rahikkala et al. reported that a novel pathogenic mutant of *SMG9* led to intellectual disability [68]. Additionally, NMD plays an important role in certain forms of cancer. Some tumors recruit NMD to downregulate tumor-suppressor mRNAs by selecting for destruction-inducing mutations, while others involve NMD factor mutations that disable NMD activity, allowing tumor cells to upregulate pathways that are normally NMD targets and favor their adaptation to the microenvironment [78,79]. NMD also participates in innate immune responses to degrade transcripts of RNA viruses in mammalian cell lines, plants, and insects [81]. Balistreri et al. reported that the ablation of NMD factors UPF1, SMG5, and SMG7 increased the amount of RNA, proteins, and titers of a positive-stranded RNA virus [75]. Recently, one study revealed that AS-NMD regulates OAS1, an antiviral protein. The expression of OAS1 is correlated with coronavirus disease 2019 (COVID-19) susceptibility [76]. The association of NMD dysregulation with a spectrum of conditions ranging from neurodevelopmental disorders to viral infections highlights its diverse impact on human health. The transcript-based annotation of RNAseq data allows for the identification of the differential expression of NMD-sensitive transcripts or isoforms [82], aiding in the assessment of NMD efficiency change and its correlation with genetic disease. As research continues to unravel the intricate mechanisms and consequences of NMD, there is great potential for leveraging this knowledge to develop targeted therapies for NMD-related disorders and to enhance our understanding of disease mechanisms at the molecular level.

### 4.3. Stress Responses

NMD is critical in stress and environmental adaptation. For example, in *S. cerevisiae*, NMD is essential in regulating magnesium homeostasis [51]. Wang et al. reported that *Upf1* deletion mutants are more resistant to elevated copper levels due to the regulation of *Ctr2* mRNA, which encodes a copper transporter of the vacuolar membrane [50]. An extensively studied stress is endoplasmic reticulum (ER) stress, which activates the unfolded protein response (UPR) intended to restore homeostasis. Sakaki et al. showed that NMD is essential for ER homeostasis. In *C. elegans*, the loss or reduced expression of *smg-1, smg-4,* and *smg-6* induce ER stress, as does defective SMG6 in HeLa cells [56]. mRNA encoding the key UPR regulator, IRE1α, has a long 3′ UTR that is an NMD target [83]. Thus, the NMD targeting of IRE1, while not completely abolishing expression, fine tunes its level to help keep the UPR inactive in the absence of ER stress. During ER stress, eIF2α is phosphorylated by PERK, which suppresses NMD by lowering translation and allowing the activation of UPR factors. Once homeostasis is restored, NMD promotes the termination of the UPR [77]. The ER stress response mechanism explains why adding the ER stress inducer, thapsigargin, to a C2C12 myogenic cell line suppresses NMD activity [84]. Similarly, hypoxic stress abrogates NMD via the phosphorylation of eIF2α [66]. Besides ER stress, amino acid starvation also downregulates NMD, again by lowering translation, which increases the stability of transcripts encoding factors that restore amino acid homeostasis [74] and the induction of autophagy that helps to recycle proteins back into amino acids [85].

### 4.4. Longevity

To date, only a few studies investigated how NMD affects longevity. NMD activity decreases during aging, but the changes vary among different tissues. Notably, research in *C. elegans* has unveiled the crucial role of NMD factors in promoting an extended lifespan in specific genetic contexts. For instance, NMD factors are essential for promoting the fully extended lifespan of *daf-2*/insulin-like receptor *C. elegans* mutants [57], highlighting the interplay between insulin signaling pathways and NMD in longevity regulation. Recently, Kim et al. also reported *algn-2*, the expression of which decreases during aging, to be essential for the normal lifespan of *C. elegans* in a NMD-dependent manner [55]. The protein ALGN-2 serves as a positive regulator of NMD. When ALGN-2 is upregulated by inhibiting the *daf-2/*insulin/IGF-1 receptor, the lifespan increases [55]. In particular, neuronal NMD was important for lifespan extension. Under DR, the prevalence of intron retention produced via alternative splicing (AS) increases concurrently with lower NMD resulting from reduced nutrient availability [16]. When knocking down NMD factors *smg-6* or *smg-7* independently via RNAi during early adulthood, the beneficial effect of DR on longevity was lost [16]. This phenotype was further confirmed with *smg-6* and *smg-7* knockout mutants (unpublished data). Another study showed that a *C. elegans eat-2* mutant, which is a genetic model of DR with reduced pharyngeal pumping, requires *smg-2* in AS-NMD for maximum increased lifespan [58]. Interestingly, the regulation of NMD on longevity under DR is not dependent on the translation-regulating integrated stress response (ISR). Using an *eif-2α* phospho-null mutant, NMD activity is still decreased under DR and AS-NMD events remain similar to wild-type N2 *C. elegans* [86]. In addition, translation can be downregulated in *eif-2α* phospho-null mutants, which explains why NMD activity is barely affected by ISR interruption [86]. Together, these studies are beginning to shed light on the important role of NMD in lifespan regulation.

## 5. Genetic Tools for Studying NMD and the Physiological Outcomes

While the thrust of NMD research focuses on understanding the molecular mechanisms underpinning its function, fewer studies address its role in governing metazoan physiology, especially with respect to tissue-specific functions. Linde et al. demonstrated that NMD is significantly more efficient in HeLa cells than MCF7 cells for multiple mRNA targets [43]. Likewise, compared to HeLa cells, NMD downregulates TCRβ transcripts more strongly in T cells [87]. In addition, inter-tissue variation in NMD efficiency was shown by Zetoune et al. [45]. Their study found that the testis, ovary, brain, and heart exhibit high NMD efficiencies to downregulate the nonsense mutation of the *MenI* gene, whereas the lung, intestine, and thymus exhibit weaker downregulation. However, it is unclear how tissue-specific variation in NMD is regulated and if different branches of NMD are involved. Another unanswered question has to do with the physiological outcomes of these variations. The limitations of in vitro studies and the relatively long lifespan of mice pose challenges to the use of these models in further exploring the link with NMD in the context of longevity. Shorter-lived, intact animal systems with a large genetic toolbox will be important to help fill the gaps in understanding the role of NMD in different tissues and the consequences for organismal physiology, especially with respect to the coordination of adaptive responses.

### 5.1. Genetic Techniques

Many genetic tools are available for studying the interactions of NMD and physiology. Two of the most potent in their ability to elucidate gene function are RNA interference (RNAi) and clustered regularly interspaced short palindromic repeats (CRISPR). 

RNAi attenuates gene expression by binding to and inducing the degradation of mRNA targets [88]. As a tool for knockdown genes, RNAi is used frequently to study gene functions in cell cultures, *C. elegans*, and *Drosophila*. Two novel NMD factors, *smgl-1* and *smgl-2*, escaped early discovery because null mutations result in early developmental lethality. They were only found because of the flexibility in timing knockdown, with respect to development stage, that is possible with RNAi screening [89]. Tabrez et al. used RNAi to demonstrate that AS-NMD regulates longevity under DR [58]. Domeier et al. reported that *smg-2* mutation in *C. elegans* was able to recover from the paralyzed phenotype of *unc-54* RNAi, indicating that the persistence of RNAi requires some *smg* genes [90]. Compared to other methods, RNAi is convenient and economical.

CRISPR-associated genome editing has been used widely in genetics research in recent years because of the precision and relative ease with which it is carried out. Shasheen et al. knocked out *smg9* in mice using CRISPR/Cas9 and showed that it is essential for normal development [67]. This approach is very convenient for exploring the role of specific genes in NMD functions and the corresponding effects on animal or cell physiology. 

### 5.2. Genetic Models

Several eukaryotic models have been developed to study the physiology related to NMD, including but not limited to cell lines, yeast, nematodes, fruit flies, plants, and mice (Table 1). To understand the interaction between NMD and physiology in a multicellular system, the invertebrate animal *C. elegans* is one of the premiere models. The maintenance of *C. elegans* is easy and cheap. The relatively short lifespan allows for the time-efficient evaluation of longevity-related phenotypes. With the availability of RNAi libraries and CRISPR techniques, impacts on physiology (e.g., lifespan, morphology, body size, progeny, stress response, and growth) can be rapidly explored [91]. *C. elegans* also allows for tissue expression and crosstalk studies, making it possible to tease apart tissue-selective NMD functions, which can be used to inform studies in more complex systems. 

Another animal model that is attracting attention in genetic research is *Nothobranchius furzeri*, commonly referred to as the African turquoise killifish. This small fresh water vertebrate has a short lifespan of only 4–6 months, with similarly rapid development, going from late-stage embryo to egg-laying adult in only 30–40 days [92]. Vertebrate-specific physiology, including different tissues and adaptive immunity, can be studied in turquoise killifish. Two genetic modification methods, Tol2 DNA transposase and the CRISPR/Cas9 system, have been developed for the fish, which offer a rapid genome-to-phenotype platform for vertebrates [93]. Until now, no research focusing on NMD has been conducted in this species. However, it is a promising animal model for understanding the life-long impact of changes in NMD function in a range of environmental and genetic contexts.

## 6. Perspectives and Concluding Remarks

NMD performs important roles in the mRNA quality control surveillance and differential gene expression critical to physiological adaptation. Different branches of NMD have been discovered over the years, but further research is needed to understand their function under different conditions and within specific tissues. Exploring how NMD activity varies among tissues in response to physiological cues or pathological conditions could shed light on disease pathogenesis and favor therapy development. NMD is generally important for growth and development, as well as adaptive responses to changing environmental conditions. While more studies are necessary to understand fully the physiological outcomes of NMD, with the advance of technology and availability of genetic tools and diverse systems, a better understanding of its role in health and disease is not out of the question. Additionally, the role of NMD in aging presents an intriguing research direction. Investigating the interplay between NMD, cellular senescence, and age-related diseases will lead to strategies promoting healthy aging and extended lifespans. Understanding how NMD dysregulation contributes to age-related pathologies could help in identifying novel intervention targets.

## Figures and Tables

**Table 1:** Physiological consequences of NMD.

Phenotypes	Species	In Vitro or In Vivo	References
Growth rates of NMD mutants is reduced on nonfermentable carbon sources	*S. cerevisiae*		[47,48]
NMD mutants are sensitive to Calcofluor white (cell wall disruptor)	*S. cerevisiae*		[49]
NMD mutants are more tolerant to toxic concentrations of copper	*S. cerevisiae*		[50]
NMD regulates magnesium homeostasis	*S. cerevisiae*		[51]
UPF1 is not essential for growth	*S. cerevisiae*		[52]
NMD mutants have abnormal morphogenesis on the genitalia and reduced offspring numbers	*C. elegans*	In vivo	[53]
NMD mutants rescued the worms from *unc-54 (r293)* movement defects	*C. elegans*	In vivo	[54]
*algn-2*, a positive regulator of NMD, is essential for longevity	*C. elegans*	In vivo	[55]
*smg-1, -4,* and *-6* defects in *C. elegans* and depletion of *SMG6* in HeLa cells cause endoplasmic reticulum stress	*C. elegans; H. sapiens*	In vitro; In vivo	[56]
NMD is required for longevity through the insulin-like signaling pathway	*C. elegans*	In vivo	[57]
NMD coupled with alternative splicing is required for longevity from dietary restriction (DR)	*C. elegans*	In vivo	[58]
*smg-6* and *smg-7* mutants showed reduced lifespan under DR	*C. elegans*	In vivo	[16]
NMD mutants cause lethality during larval development	*Drosophila*	In vivo	[59]
NMD is essential for zebrafish embryonic development and survival	*D. rerio*	In vivo	[60]
RENT1/UPF1, UPF2, SMG1, and SMG6 are essential for mammalian embryonic viability	*M. musculus*	In vitro; In vivo	[61,62,63,64]
Deletion of UPF2 led to extinction of hematopoietic stem and progenitor cells	*M. musculus*	In vivo	[64]
Upf2 ablation leads to testicular atrophy and male sterility in embryonic Sertoli cells	*M. musculus*	In vitro	[65]
NMD is required in hypoxic stress response	*M. musculus*	In vitro	[66]
SMG9 mutation causes a multiple congenital anomaly syndrome and intellectual disability	*H. sapiens; M. musculus*	In vivo	[67,68]
NMD involves in specifying the developmental fate of embryonic stem cells	*H. sapiens*	In vitro	[69]
Mutation in *UPF3B* cause intellectual disability (ID); Mutation in *UPF2* is associated with ID and neuro-developmental disorders	*H. sapiens*	In vivo	[70,71,72,73]
NMD is inhibited by amino acid starvation and transcripts that promote amino acid homeostasis is upregulated	*H. sapiens*	In vitro	[74]
NMD has antiviral activity	*H. sapiens*	In vitro	[75]
NMD affects COVID-19 susceptibility via regulating OAS1 expression	*H. sapiens*	In vitro; In vivo	[76]
NMD is downregulated to restore homeostasis under endoplasmic reticulum stress	Mammals	Review	[77]
In cancer, some types of tumors use NMD to downregulate tumor-suppressor mRNAs by selecting for destruction-inducing mutations; other types of tumors disable NMD by NMD factor mutations, which favors the tumor cells to adapt to microenvironment	Mammals	Review	[78,79]
